# The *C. elegans* miR-235 regulates the toxicity of graphene oxide via targeting the nuclear hormone receptor DAF-12 in the intestine

**DOI:** 10.1038/s41598-020-73712-x

**Published:** 2020-10-09

**Authors:** Tiantian Guo, Lu Cheng, Huimin Zhao, Yingying Liu, Yunhan Yang, Jie Liu, Qiuli Wu

**Affiliations:** 1 Institute of Nephrology, Zhong Da Hospital, Medical School, Southeast University, Nanjing, China; 2 Monash Biomedicine Discovery Institute and Department of Anatomy and Developmental Biology, Monash University, Melbourne, VIC 3800 Australia

**Keywords:** Nanotoxicology, Environmental sciences, Non-coding RNAs

## Abstract

The increased application of graphene oxide (GO), a new carbon-based engineered nanomaterial, has generated a potential toxicity in humans and the environment. Previous studies have identified some dysregulated microRNAs (miRNAs), such as up-regulated *mir-235*, in organisms exposed to GO. However, the detailed mechanisms of the dysregulation of miRNA underlying GO toxicity are still largely elusive. In this study, we employed *Caenorhabditis elegans* as an in vivo model to investigate the biological function and molecular basis of *mir-235* in the regulation of GO toxicity. After low concentration GO exposure, *mir-235* (*n4504*) mutant nematodes were sensitive to GO toxicity, implying that *mir-235* mediates a protection mechanism against GO toxicity. Tissue-specific assays suggested that *mir-235* expressed in intestine is required for suppressing the GO toxicity in *C. elegans*. *daf-12*, a gene encoding a member of the steroid hormone receptor superfamily, acts as a target gene of *mir-235* in the nematode intestine in response to GO treatment, and RNAi knockdown of *daf-12* suppressed the sensitivity of *mir-235*(*n4503*) to GO toxicity. Further genetic analysis showed that DAF-12 acted in the upstream of DAF-16 in insulin/IGF-1 signaling pathway and PMK-1 in p38 MAPK signaling pathway in parallel to regulate GO toxicity. Altogether, our results revealed that *mir-235* may activate a protective mechanism against GO toxicity by suppressing the DAF-12-DAF-16 and DAF-12-PMK-1 signaling cascade in nematodes, which provides an important molecular basis for the in vivo toxicity of GO at the miRNA level.

## Introduction

Graphene oxide (GO), a novel engineering nanomaterial composed of carbon atoms with surface-rich functional groups, has been widely used in various areas owing to its excellent physicochemical characteristics^1–5^. Meanwhile, effect of GO exposure to human and environmental animals has aroused extensive attention with its application. It has been reported that GO exposure can induce toxicity including high level of reactive oxygen species (ROS), cell apoptosis and inflammatory in vitro and in vivo^6–9^. However, the molecular mechanisms of organisms response to GO toxicity are largely unknown.

*Caenorhabditis elegans* (*C. elegans*) is a powerful model animal for genetic investigation of various biological processes^10^. Owing to its sensitivity to environmental toxicants, *C. elegans* has also been widely used as an in vivo model for studying the toxicity assessment and toxicological mechanisms of environmental toxicants^11^. Previous studies have found that GO exposure causes a short lifespan, attenuate athletic ability, reduced reproductive capacity and increased intestinal ROS in *C. elegans*^7^. Meanwhile, several important molecular signaling pathways including c-Jun N-terminal kinase (JNK), p38 MAP Kinase (MAPK), insulin growth factor-1 (IGF-1), transforming growth factor-β (TGF/β), Wnt, oxidative stress associated, apoptosis, and DNA damage signaling pathways have been identified to be involved in regulating GO toxicity in nematodes^12–17^.

Recent studies have indicated that some microRNAs (miRNAs) may function in the control of GO toxicity, which further improves our understanding of the molecular mechanism of GO toxicity^18–20^. miRNAs are a class of endogenous nucleotide non-protein-encoding RNAs with 21–23 bases, and regulate eukaryotic gene expression at the post-transcriptional level^21,22^. miRNAs inhibit gene expression primarily by binding to certain sites of the 3′ untranslated regions (3′ UTRs) of target mRNAs, which results in degradation of mRNA and inhibition of protein translation^23–25^. In *C. elegans*, miRNAs also play important roles in response to the toxicity of certain carbon nanomaterials. For example, *let-7*, *mir-259*, *mir*-*35*, and *mir-355* are involved in regulating the multi-walled carbon nanotubes (MWCNTs) toxicity in *C. elegans*^26–29^. Moreover, *let-7* acted as a downstream target for epidermal BLI-1 in the regulation of GO-PEG toxicity^20^.

In previous studies, we have found several dysregulated miRNAs in GO exposed nematodes, and tested the function of candidate miRNAs in regulating GO toxicity^30^. Among these dysregulated miRNAs, *mir-235* was up-regulated in GO-exposed nematodes. *mir-235*, a sole orthologue of mammalian miR-92 in oncogenic miR-17-92 cluster, acts in the hypodermis and glial cells to arrest postembryonic developmental events in neuroblasts and mesoblasts^31^. *mir-235* is induced by dietary restriction at the end of larval development, which subsequently suppresses Wnt signaling by inhibiting cwn-1/WNT4 and thereby promotes longevity^32^. Our previous study found that *mir-235*(*n4504*) mutants display a sensitive property to GO toxicity^30^. However, it is still unclear about the molecular basis of *mir-235* in response to the GO toxicity at low concentration.

In this study, we investigated the molecular mechanisms of *mir-235* in regulating GO toxicity using the in vivo assay system of *C. elegans*. We found that *mir-235* regulates GO toxicity via targeting *daf-12* in the intestine. The nuclear hormone receptor DAF-12, a homolog of vertebrate vitamin D and liver X receptors, functions as a ligand-dependent switch that regulates the developmental progression and arrest in response to environmental cues^33–35^. Our results indicated that the intestinal *mir-235/*DAF-12 acted the upstream of both DAF-16 in the insulin/IGF-1 and PMK-1 in p38 MAPK signaling pathway in parallel to regulate GO toxicity in nematodes.

## Results

### Physicochemical properties of prepared GO

The ultrasound-treated GO was a single layer of nanosheet, the thickness of which was approximately 1.0 nm based on AFM assays (Fig. S1A). After sonication, the size distribution of most GO was in the range of 40–50 nm (Fig. S1B). Raman spectroscopy assay showed that GO had a D band (1354.99 cm^−1^) and a G band (1599.04 cm^−1^), respectively (Fig. S1C). The D band reflected the disorder of graphite layer introduced after treatment with sulfuric acid and KMnO_4_. Zeta potential of GO in K medium was − 22.3 ± 2.5 mV.

### *mir-235* acts in the intestine to control GO toxicity

Our previous studies have suggested that the mutation of *mir-235* induced a sensitive property to GO concentration of 10 mg/L in *C. elegans*^30^. However, most nanomaterials released into the environment are in the range of ng/L or μg/L^36,37^. To further determine the function of *mir-235* in response to GO toxicity at the low-concentration, we quantified the ROS production and locomotion behavior of *mir-235*(*n4504*) mutant nematodes exposed to 100 μg/L GO. After the prolonged exposure from L1-larvae for 96 h, we found that *mir-235* mutant nematodes generate more ROS and display a decreased locomotive speed, compared with control animals, which suggests that *mir-235* mutant nematodes still sensitive to GO exposure (Fig. S2).

**Figure 1:**
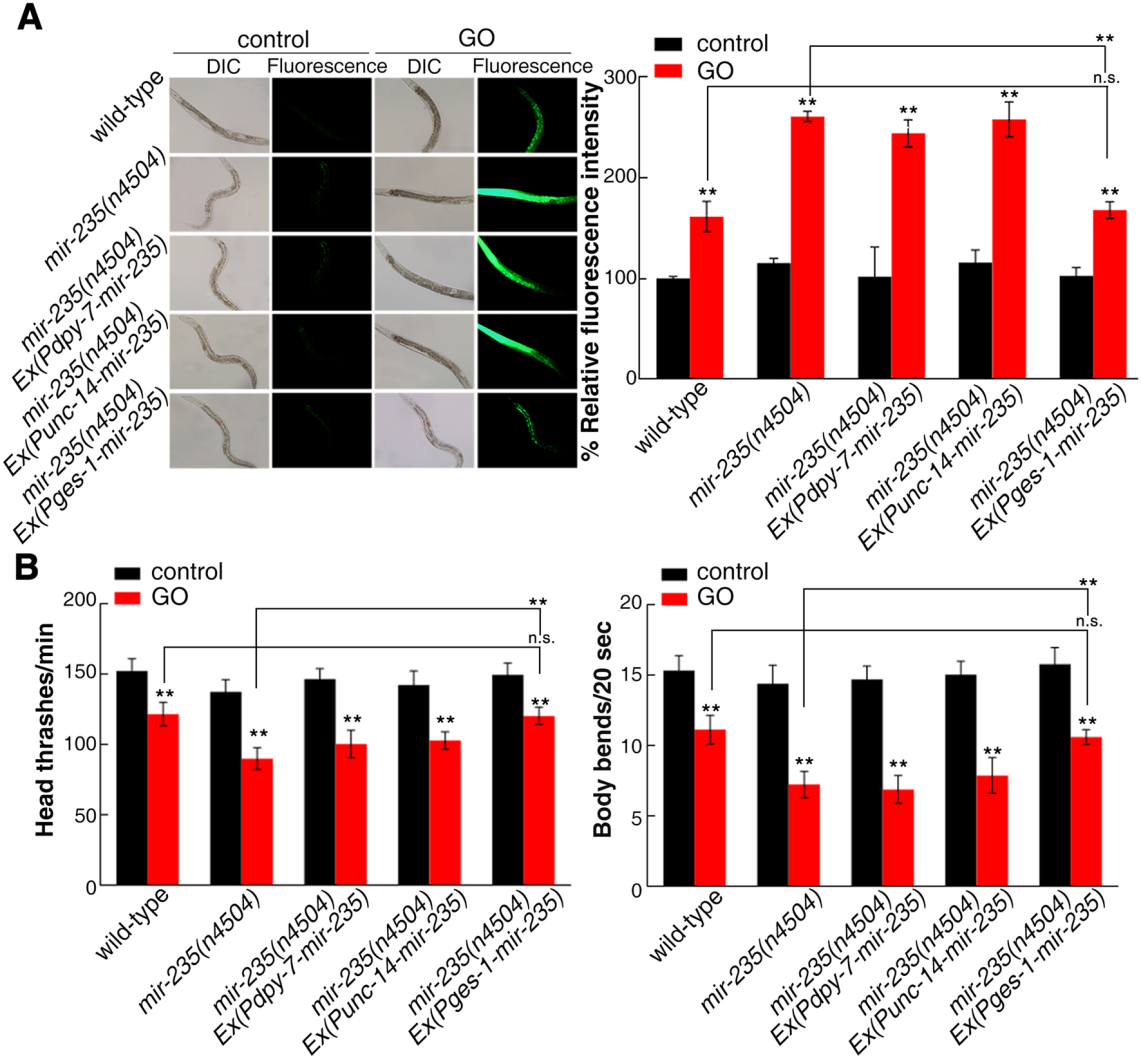
Tissue-specific activity of *mir-235* in regulating GO toxicity. (**A**) Tissue-specific activity of *mir-235* in regulating GO toxicity on ROS production. (**B**) Tissue-specific activity of *mir-235* in regulating GO toxicity on locomotion behavior in nematodes. Bars represent means ± SD. n.s. represents no significant difference. **p < 0.01 versus control (if not specially indicated).

As *mir-235* is expressed in intestine, epidermis, and neurons^31^, we used the tissue-specific promoter to investigate the tissue-specific activity of *mir-235* in the regulation of GO toxicity. Rescue assay by expression of *mir-235* in the neurons or epidermis with the *unc-14* or *dpy-7* promoter did not significantly affect the sensitive property of *mir-235*(*n4504*) mutant to GO toxicity (Fig. 1). In contrast, the expression of *mir-235* under the control of the intestine-specific *ges-1* promoter significantly decreased the intestinal ROS production and increased locomotion behavior in *mir-235*(*n4504*) mutant exposed to GO (Fig. 1). These results indicated that *mir-235* may act in the intestine to mediate a protection mechanism against GO toxicity in nematodes.

### Intestinal candidate targeted genes of *mir-235* after GO exposure

To identify molecular targets of *mir-235* in the regulation of GO toxicity, we predicted the possible targeted genes of *mir-235* by TargetScan database. In view of *mir-235* function in the intestine to control GO toxicity, 50 target genes expressed in the intestine were further screened out from 194 possible targeted genes of *mir-235* (Table S1).

**Figure 2:**
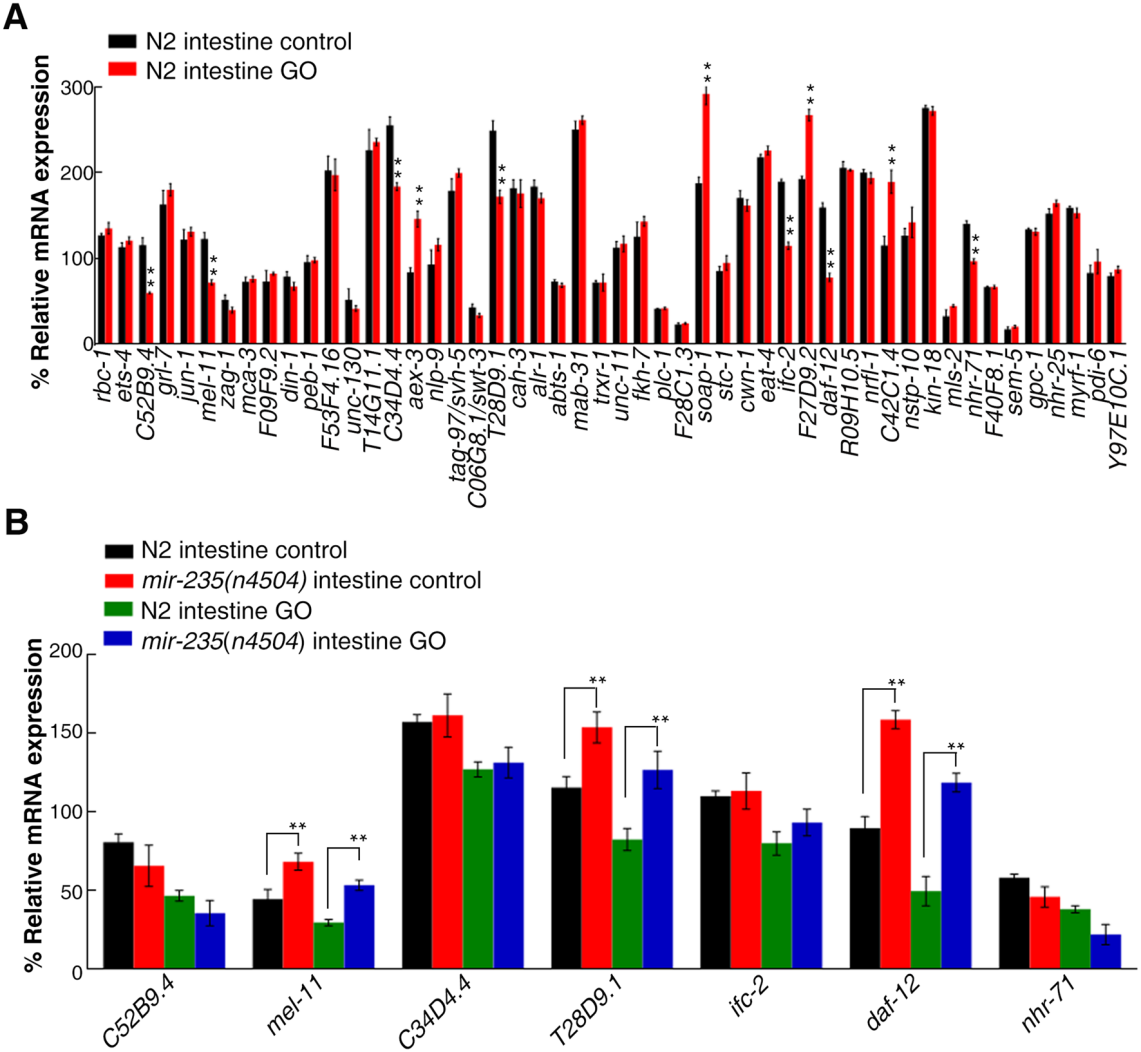
Validation of candidate intestinal targets of *mir-235* in GO exposure nematodes *via* qRT-PCR analysis. (**A**) Dysregulated candidate intestinal targets of *mir-235* in intestinal tract of wild-type nematodes exposed to GO. (**B**) Dysregulated candidate intestinal targets of *mir-235 *in intestinal tract of *mir-235* mutant nematodes exposed to GO. Bars represent means ± SD. **p < 0.01. Triplicate independent experiments were performed for the assays.

To identify the *mir-235* targeted genes in *C. elegans* intestine, we isolated the intestine and extracted the RNA as previously reported^39^. Our genetic assays of 50 predicted genes showed that the expression levels of *C52B9.4*, *mel-11*, *C34D4.4*,* T28D9.1*, *ifc-2*, *daf-12* and *nhr-71* were decreased, and the expression levels of *aex-3*, *soap-1*, *F27D9.2* and *C42C1.4* were increased in wild-type N2 intestine after GO exposure (Fig. 2A). Considering the increased expression of *mir-235* in wild-type N2 exposed to GO, we further focus on these genes with decreased expression after GO exposure in the intestine of wild-type N2. We found that the expression levels of *mel-11*, *T28D9.1* and *daf-12* were increased in the intestine of *mir-235*(*n4504*) mutant compared with that in the intestine of wild-type N2 (Fig. 2B). Therefore, the results suggest that these 3 genes (*mel-11*, *T28D9.1* and *daf-12*) may be candidate targets for *mir-235* in the intestine*.*

### Intestinal *daf-12* is a potential *mir-235* target in the regulation of GO toxicity

**Figure 3:**
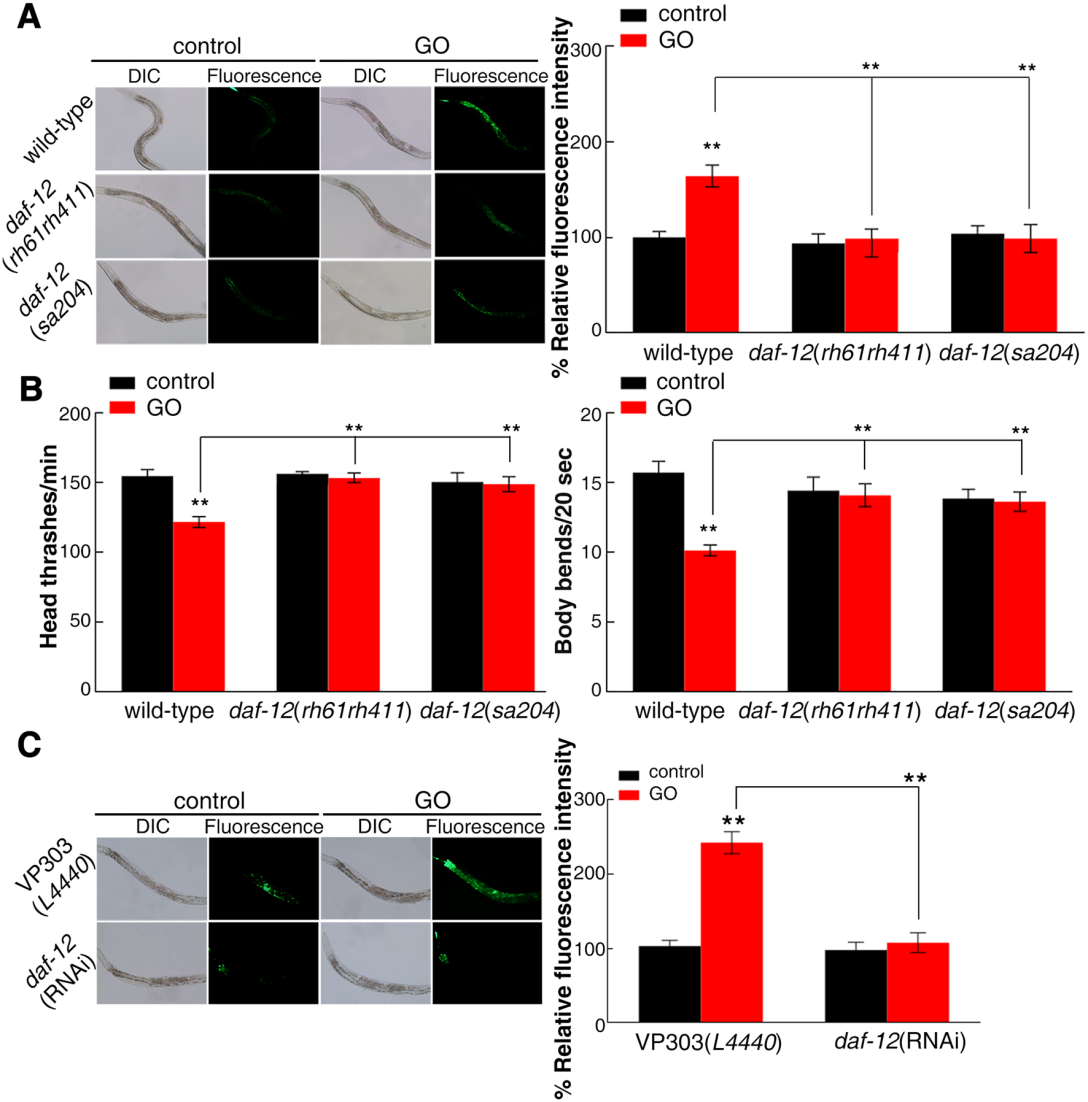
Effects of *daf-12* mutation or intestine-specific RNAi knockdown on GO toxicity. (**A**) Effect of *daf-12* mutation on GO toxicity in decreasing ROS production. (**B**) Effect of *daf-12* mutation in regulating GO toxicity in increasing locomotion behavior. (**C**) Intestine-specific activity of *daf-12* in regulating GO toxicity in decreasing ROS production. Bars represent means ± SD. **p < 0.01. Number of nematodes is 25–30 per condition, and the experiment was repeated three times independently.

*daf-12* encodes a member of the steroid hormone receptor superfamily homologous to human vitamin D receptor^33–35^. Among the 3 candidate target genes analyzed above, the expression level of *daf-12* was increased most significantly in *mir-235*(*n4504*) exposed to GO, which implied that *daf-12* plays a key role in response to GO toxicity (Fig. 2B). Therefore, we analyzed the effect of GO exposure on the intestinal ROS production and locomotion endpoints of *daf-12*(*rh61rh411*) and *daf-12*(*sa204*) mutants. After GO exposure, we found that *daf-12*(*rh61rh411*) and *daf-12*(*sa204*) mutants caused the resistance of nematodes to GO toxicity in inducing ROS production and in decreasing locomotion behavior (Fig. 3A,B).

Because *mir-235* acts in the intestine to regulate GO toxicity in nematodes and *daf-12* gene is also expressed in the intestine^38^, we next focus on whether intestinal *daf-12* directly respond to GO toxicity. Using intestine-specific interference nematodes (VP303), we found that intestinal-specific RNAi knockdown of *daf-12* significantly inhibited GO toxicity in inducing intestinal ROS production (Fig. 3C). That is, *daf-12* acts in the intestine to regulate GO toxicity in nematodes, which also implies that *daf-12* is a potential target for *mir-235* in regulating GO toxicity in the intestine of nematodes.

### In vivo 3′ UTR binding assay of *daf-12* with* mir-235*

**Figure 4:**
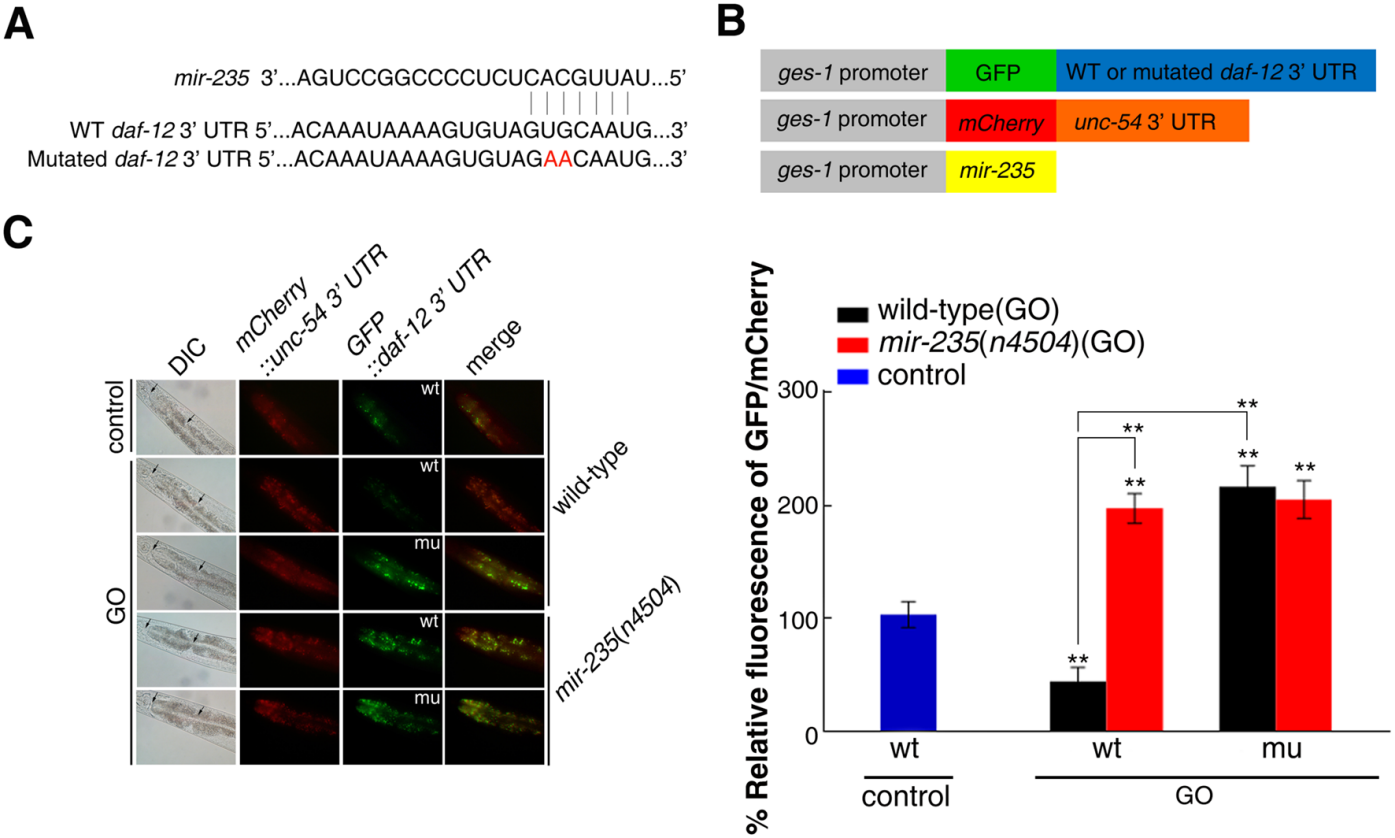
In vivo 3′ UTR binding assay of *daf-12 *with *mir-235*. (**A**) Predicted binding site on *daf-12* 3′ UTR by Targetscan. Mutated nucleotides for experiments are indicated in red. WT, wild-type. (**B**) DNA construct outline. (**C**) Fluorescence images of the *daf-12* 3′ UTR GFP reporter in nematodes exposed to GO. The fluorescence intensity of the first two pairs of intestinal cells was quantified (black arrows indicated quantitative area in DIC images). wt: wild-type. mu: mutation of the putative binding site for *mir-235* in *daf-12* 3′ UTR. Bars represent means ± SD. **p < 0.01. Number of nematodes is 25–30 per condition, and the experiment was repeated three times independently.

To further confirm whether *daf-12* is a direct target of *mir-235*, we firstly predicted the binding site of *daf-12* 3′ UTR with *mir-235* by Targetscan. It suggested that *daf-12* 3′ UTR had 7 bases complementary to *mir-235* (Table S2 and Fig. 4A). We then constructed transgenic nematodes which contain a GFP vector driven by intestinal-specific *ges-1* promoter and GFP reporter under the control of the *daf-12* 3′ UTR (wild-type or *mir-235* binding site mutated from GUGCAAU to GAACAAU) (Fig. 4A,B). P*ges-1*::mCherry *unc-54* 3′ UTR was used as a control because *mir-235* can not bind to *unc-54* 3′ UTR. We found that the expression of *daf-12* GFP in the intestine was significantly reduced after GO exposure in wild-type nematodes with *daf-12* 3′ UTR (wild-type) (Fig. 4C). However, mutation of the putative binding site for *mir-235* in *daf-12* 3′ UTR abolished the reduction of GFP expression in wild-type nematodes (Fig. 4C) After GO exposure, the GFP expression of *daf-12* 3′ UTR (wild-type) was significantly increased in *mir-235*(*n4504*) nematodes than that in wild-type nematodes (Fig. 4C). Thus, our analysis further supports that intestinal *daf-12* is a direct targeted gene of *mir-235* in regulating the response to GO toxicity.

### Genetic interaction between *mir-235* and *daf-12* in regulating the response to GO toxicity

**Figure 5:**
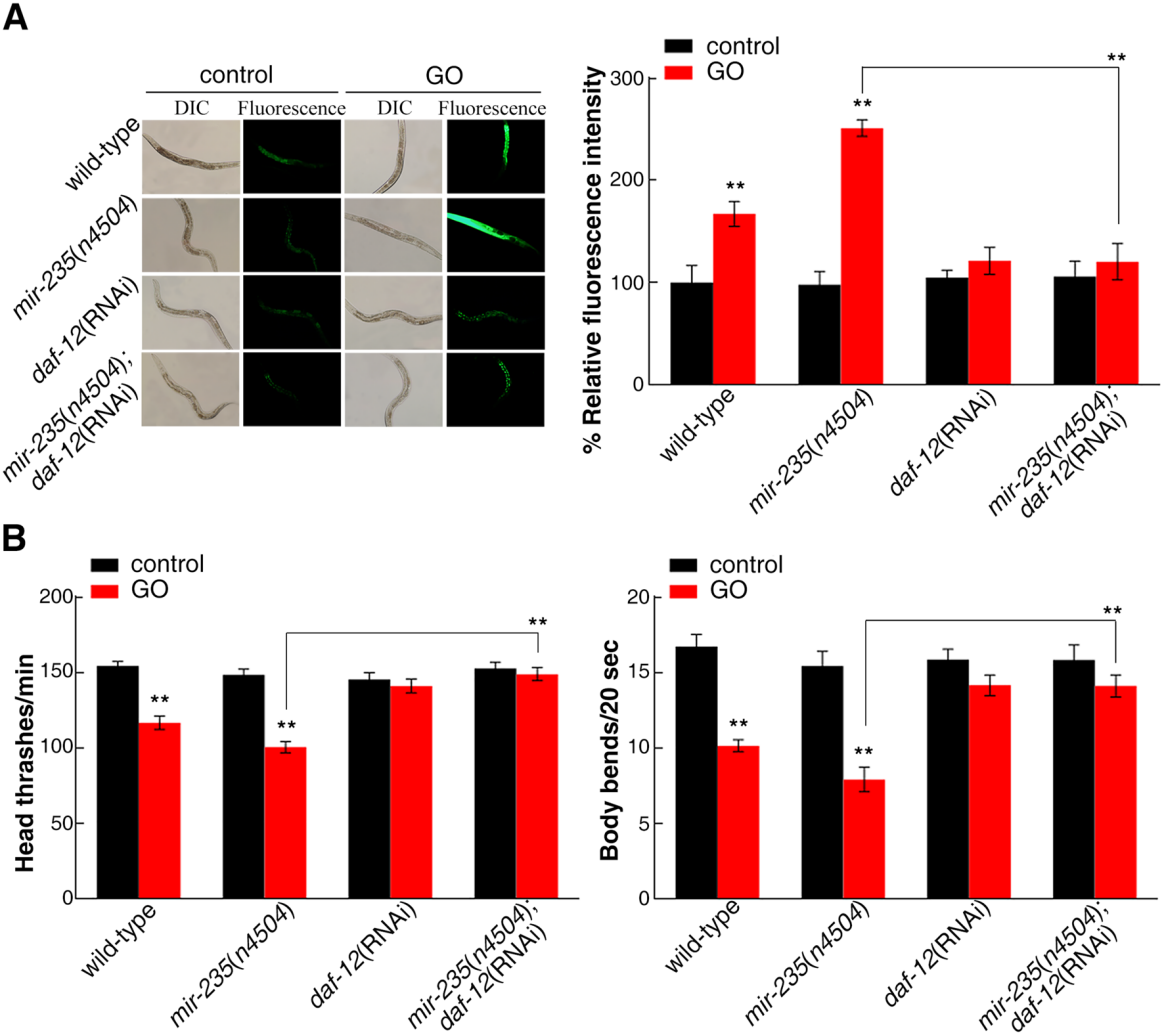
Genetic interactions between *mir-235* and *daf-12* in regulating GO toxicity. (**A**) Genetic interactions between *mir-235* and *daf-12* in regulating GO toxicity in decreasing ROS production. (**B**) Genetic interaction between* mir-235* and *daf-12* in regulating GO toxicity in increasing locomotion behavior in nematodes. Bars represent means ± SD. **p < 0.01. Thirty nematodes were examined per treatment, and the experiment was repeated three times independently.

To further investigate the genetic interaction between *mir-235* and *daf-12* in the regulation of GO toxicity, we used *daf-12* RNAi strain to interfere with *mir-235*(*n4504*) mutant to obtain nematodes *mir-235*(*n4504*); *daf-12*(RNAi)*.* After exposure to GO, *mir-235*(*n4504*); *daf-12*(RNAi) showed the similar phenotype with *daf-12* RNAi knockdown nematodes in decreasing ROS production and increasing locomotion behavior (Fig. 5), which indicated that *daf-12* RNAi knockdown suppressed the susceptibility of *mir-235* mutant nematodes. Therefore, *daf-12* serves as a downstream gene of *mir-235*, and regulates GO toxicity by inhibiting the function of *mir-235.*

### Effect of *mir-235* and *daf-12* on the distribution of GO in nematodes

**Figure 6:**
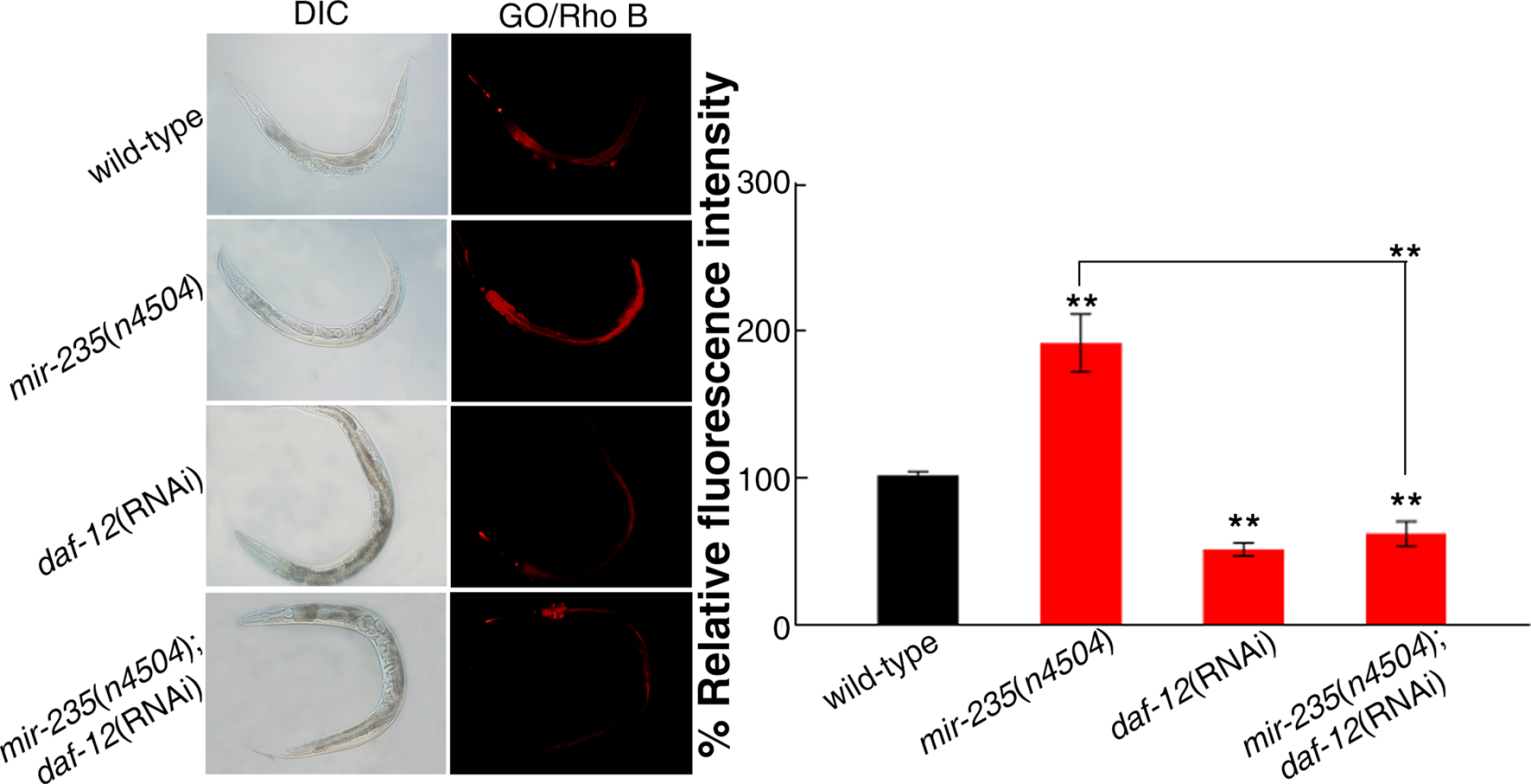
Distribution and translocation of GO/Rho B in nematodes. Bars represent means ± SD. **p < 0.01. Between 25 and 30 nematodes were examined per condition, and the experiment was repeated three times independently.

Distribution and translocation of GO are key factors of the toxicity formation in vivo^40^. Previous study has showed that the fluorescent molecular probe Rho B can interact with GO because of its π–π stacking^41^. By using UV–Vis to monitor the residual amount of Rho B in the solution after the loading process, it was found that almost 95% of the Rho B was loaded on the GO film^41^. At present, GO/Rho B has been used to reflect the distribution and translocation of GO in cells and in organisms^40,42^. Therefore, we used Rho B to label GO, which allows us to visualize the distribution of GO in nematodes. After GO/Rho B exposure, we found that only a small amount of GO was transported into the intestine in wild-type animals. In sharp contrast, *mir-235*(*n4504*) mutant nematodes have more GO in intestine (Fig. 6). Interestingly, RNAi knockdown of *daf-12* in either wild type or *mir-235*(*n4504*) significantly reduced the intestinal distribution and translocation of GO (Fig. 6). These data indicated that the *mir-235* mutation significantly enhanced the distribution and translocation of GO in nematodes, and RNAi knockdown of *daf-12* suppressed distribution and translocation of GO in *mir-235*(*n4504*) mutant nematodes.

### *mir-235*/DAF-12 regulated GO toxicity in the intestine by mediating the insulin/IGF-1 and p38 MAPK signaling pathways

**Figure 7:**
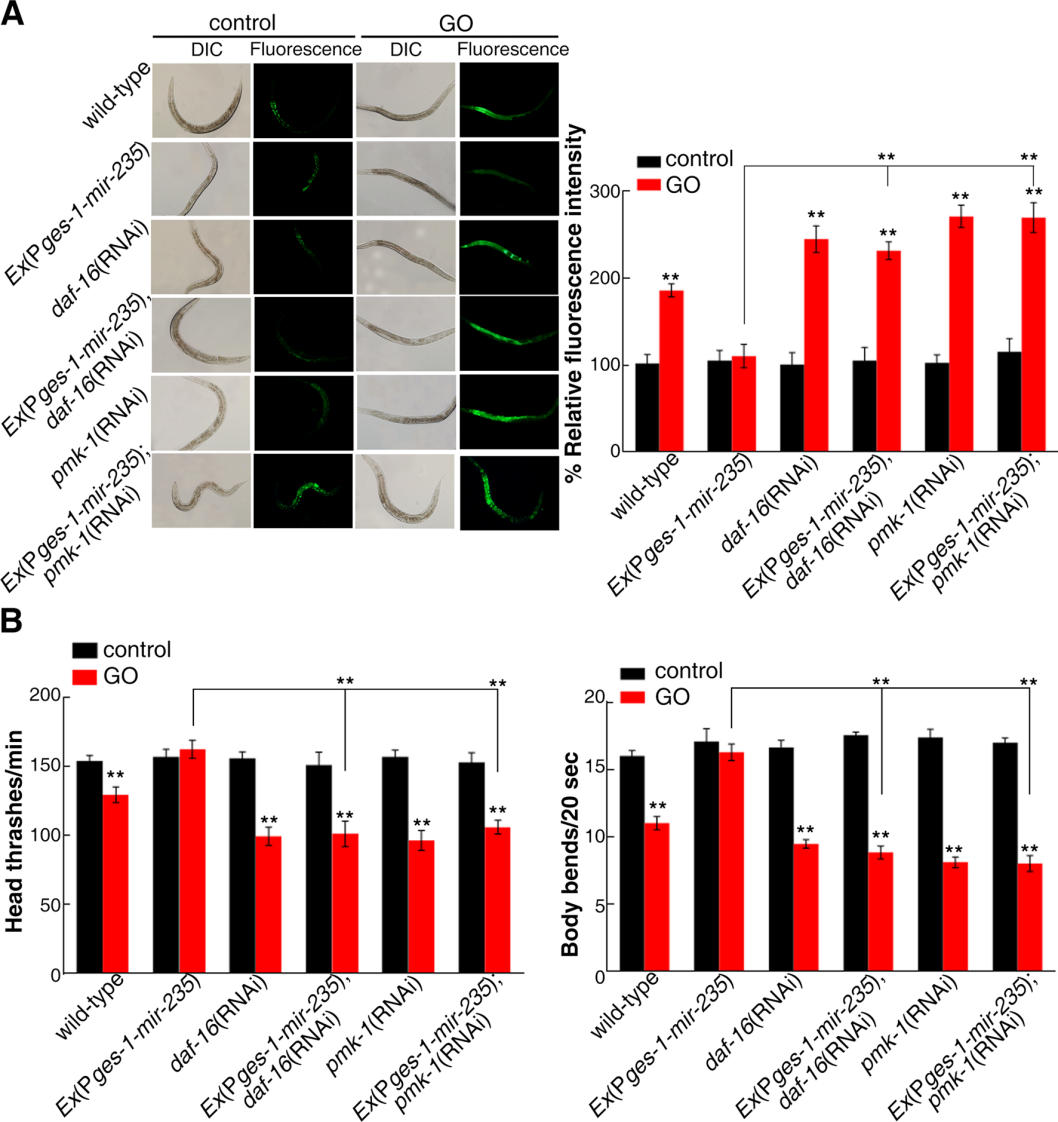
Genetic interactions between *mir-235* and *daf-16* or *pmk-1* in regulating GO toxicity. (**A**) Genetic interactions between *mir-235* and *daf-16* or *pmk-1* in regulating GO toxicity in inducing ROS production. (**B**) Genetic interaction between *mir-235* and *daf-16* or *pmk-1* in regulating GO toxicity in decreasing locomotion behavior in nematodes. Bars represent means ± SD. **p < 0.01. Thirty nematodes were examined per condition, and triplicate independent experiments were performed.

Previous study has indicated that insulin/IGF-1 and p38 MAPK signaling pathways in the intestine regulate GO toxicity in *C. elegans*^13,15^. *daf-16* gene encodes the transcriptional factor DAF-16/FOXO in the insulin signaling pathway^43^. PMK-1, as an ortholog of human MAPK14, exhibits MAP kinase activity and transcription factor binding activity in p38 MAPK signaling pathways^13^. To investigate whether *mir-235* regulates GO toxicity through insulin/IGF-1 and p38 MAPK signaling pathways, we explored the genetic interaction between *mir-235* and *daf-16* or *pmk-1* in response to GO toxicity in nematodes. We observed that RNAi knockdown of *daf-16* induced a sensitive property to GO toxicity in inducing ROS production and in decreasing locomotion behavior. Importantly, after GO exposure, the intestinal ROS production and the locomotion behavior in *Ex*(P*ges-1-mir-235*); *daf-16*(RNAi) nematodes were similar to those in *daf-16* (RNAi) nematodes, which indicated that RNAi knockdown of *daf-16* suppressed the resistance of *mir-235* intestinal overexpression nematodes to the GO toxicity (Fig. 7). This result showed that *mir-235* may act upstream of *daf-16* to regulate GO toxicity in nematodes. Similarly, we also observed that RNAi knockdown of *pmk-1* induced a sensitive property to GO toxicity and suppressed the resistance of *mir-235* intestinal overexpression nematodes to the GO toxicity (Fig. 7). This result showed that *mir-235* may also act upstream of *pmk-1* to regulate GO toxicity in nematodes.

**Figure 8:**
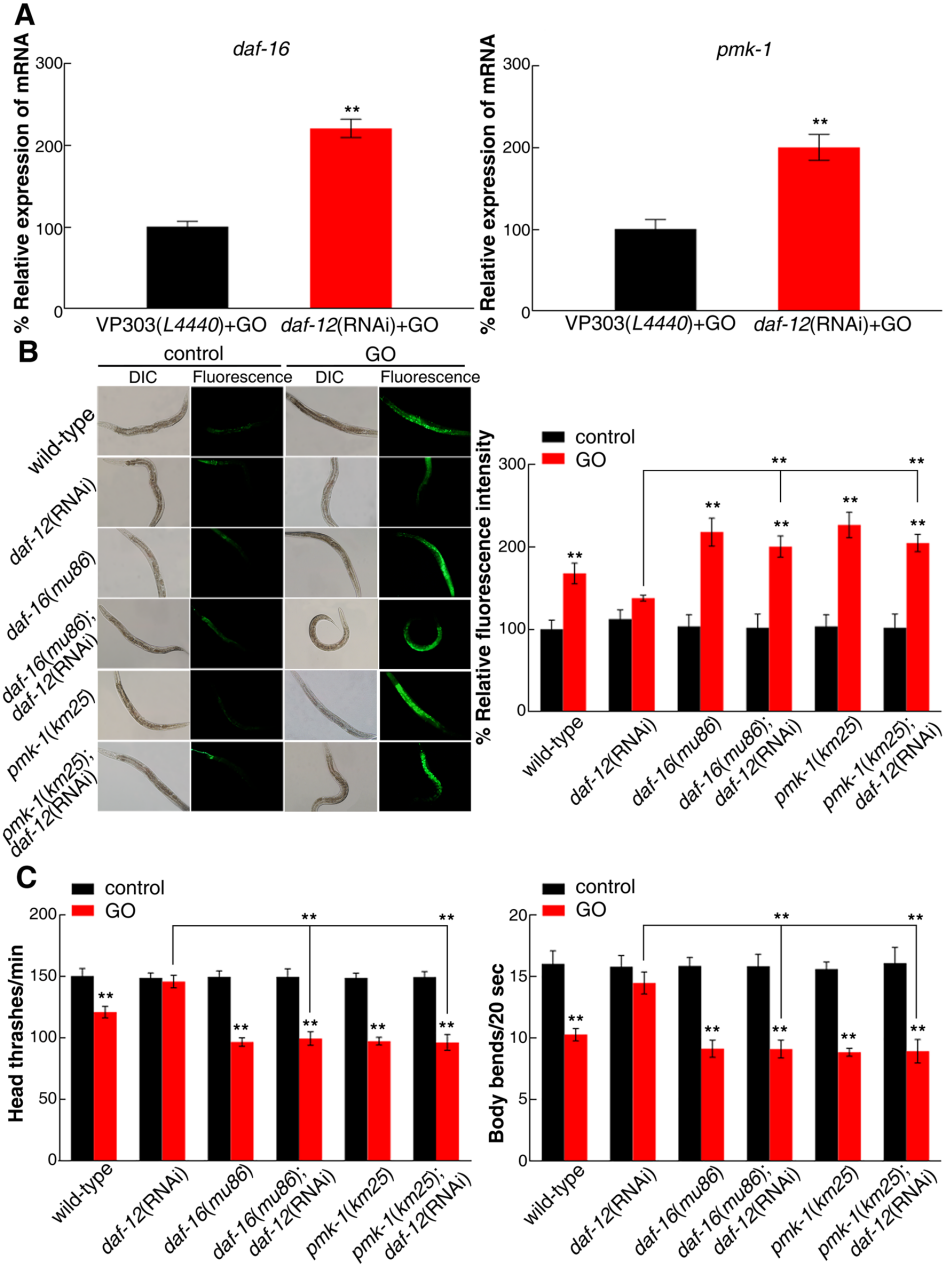
Genetic interactions between *daf-12* and *daf-16* or *pmk-1* in regulating GO toxicity. (**A**) Effects of *daf-16 *and* pmk-1* expression in *daf-12* RNAi knockdown nematodes exposed to GO *via* qRT-PCR. (**B**) Genetic interactions between *daf-12* and *daf-16* or *pmk-1* in regulating GO toxicity in inducing ROS production. (**C**) Genetic interaction between *daf-12* and *daf-16* or *pmk-1* in regulating GO toxicity in decreasing locomotion behavior in nematodes. Bars represent means ± SD. **p < 0.01. Thirty nematodes were examined per condition, and the experiment was repeated three times independently.

Considering intestinal *daf-12* as a target of *mir-235* to regulate GO toxicity, we further analyzed whether intestinal *daf-12* also regulated GO toxicity through insulin and p38 MAPK signaling pathway. After GO exposure, intestinal-specific RNAi knockdown of *daf-12* significantly increased the *daf-16* and *pmk-1* expression based on the qRT-PCR results (Fig. 8A). In contrast, there was no significant change in the expression of *daf-16* and *pmk-1* in intestinal-specific RNAi nematodes of *daf-12* without GO exposure (Fig. S3). Further, we analyzed the subcellular localization of the DAF-16::GFP fusion protein and found that RNAi of *daf-12* not only increased the expression of DAF-16::GFP, but also enhanced the translocation of DAF-16::GFP into the nucleus of intestinal cells in GO exposed nematodes (Fig. S4).

Moreover, we knockdowned *daf-12* with RNAi within *daf-16*(*mu86*) mutants to investigate the genetic interaction between *daf-12* and *daf-16* in the regulation of GO toxicity. The phenotype of *daf-16*(*mu86*); *daf-12*(RNAi) was similar to that of *daf-16*(*mu86*) mutants, based on the quantification of ROS production and locomotion behavior after GO exposure (Fig. 8B,C). Meanwhile, we also observed the phenotype of *pmk-1*(*km25*); *daf-12*(RNAi) was similar to that of *pmk-1*(*km25*) mutants (Fig. 8B,C). These results suggest that *daf-12* may play a role in regulating GO toxicity through acting upstream of *daf-16* or *pmk-1*.

To explain whether there was an interaction between *daf-16* and *pmk-1* in regulating GO toxicity, we next constructed *daf-16*(RNAi); *pmk-1*(RNAi) nematodes. We observed that *daf-16* and *pmk-1* knockdown nematodes induced a susceptibility to GO toxicity in inducing intestinal ROS production and in decreasing locomotion behavior. By contrast, *daf-16*(RNAi); *pmk-1*(RNAi) nematodes were more susceptible to the GO toxicity than *daf-16*(RNAi) or *pmk-1*(RNAi) nematodes respectively (Fig. S5), indicating that two signaling pathways acted in parallel to regulate GO toxicity. Altogether, these findings implied that *mir-235*/DAF-12 may mediate the insulin/IGF-1 and p38 MAPK signaling pathways in parallel to regulate GO toxicity in the intestine of nematodes.

## Discussion

In *C. elegans,* miRNAs have been shown to participate in the regulation of metabolic processes, cell development and lifespan determination^31,32^. Increasing evidence suggests that some miRNAs play critical roles in response to the toxicity of nanomaterials^20,30^. For example, the signaling cascade of BLI-1-let-7-HBL-1/LIN-41 is required in regulating GO-PEG toxicity. Moreover, GO-exposed *mir-244* and *mir-235* mutations induced the susceptibility to GO toxicity in decreasing lifespan. In contrast, GO-exposed *mir-247/797*, *mir-73/74* and *mir-231* mutations induced the resistance to GO toxicity in increasing lifespan. In this study, using prolonged exposure from L1 for 96 h, we found that *mir-235* mutant nematodes were sensitive to 100 μg/L GO exposure in inducing intestinal ROS production and in decreasing locomotion behavior (Fig. S2), suggesting that prolonged exposure to GO at environmentally relevant concentration induces a *mir-235*-mediated response in nematodes.

miRNA, depending on the specific tissue in which it is expressed, has important functions in various biology process, such as development, longevity and toxicity of nanomaterials^20,29,31^. In *C. elegans*, miRNA expression profiling can be determined according to the miRNA driving GFP expression^44^. As a result, *mir-235* expresses in intestine, epidermis, and neurons^31^. Previous studies also showed that *mir-235* expresses in the hypodermic, which non-autonomously regulate P and M blast cells^31^. Here, we found that the *mir-235* rescue in the intestine significantly inhibits the susceptibility of the *mir-235* mutant to GO toxicity (Fig. 1), while the rescue of *mir-235* expression in the epidermis and neurons showed a phenotype similar to that of *mir-235* mutant to GO toxicity (Fig. 1). Therefore, *mir-235* acted specifically in the intestine to regulate GO toxicity.

miRNAs can target hundreds of transcripts to regulate diverse biological pathways and processes. miRNAs degrade the target mRNA or inhibit the translation of target mRNA by complementing with the 3′ UTR of the target mRNA^45^. Studies have shown that *nhr-91* is a targeted gene of *mir-235* that involved in the regulation of development^31^. Besides, Hedgehog-related genes *grl-5* and *grl-7* are targets of *mir-235* that contribute to reactivation of quiescent neuroblasts^46^. Moreover, a variety of miRNAs have been found to bind target genes to regulate the toxicity of nanomaterials in *C. elegans.* For example, *mir-231* has been proven to regulate GO toxicity via targeting *smk-1* gene in *C. elegans*^19^. *mab-3* serves as a target for intestinal *mir-35* in regulating the response to MWCNTs^29^. In this study, we found that the expression of *daf-12* in the intestine of *mir-235* mutant, after GO exposure, was significantly increased compared with that in the intestine of wild-type N2 (Fig. 2). Both *mir-235* and *daf-12* play a role in regulating GO toxicity in the intestine (Fig. 1 and Fig. 3C). We have also proved that *mir-235* can be combined with intestinal *daf-12* 3′ UTR (Fig. 4). Moreover, RNAi knockdown of *daf-12* can reverse the susceptibility phenotype of the *mir-235* mutant to GO toxicity (Fig. 5A,B). All together, our results support that *daf-12* may be a direct target gene of *mir-235* in the intestine in response to GO toxicity.

*daf-12* encodes a nuclear receptor that regulates the dauer diapause and developmental age in *C. elegans*^38^. For example, *daf-12* can activate *let-7* miRNA, thereby regulating the developmental process through downstream target *hlb-1*^47^*. daf-16* is the downstream molecular of *daf-12* and regulates the lifespan of *C. elegans*^48^*.* Previous studies have shown that insulin and p38 MAPK signaling pathways in the intestine are involved in the regulation of GO toxicity^13,15^. In addition, genome-wide microarray analysis indicated that PMK-1 and DAF-16 form parallel pathways to promote immunity in *C. elegans*^49^. In this study, the genetic interaction analysis showed that *daf-16* and *pmk-1* also acted in parallel on the downstream of *daf-12* to inhibit GO toxicity in nematodes (Fig. 8 and Fig. S5).

**Figure 9:**
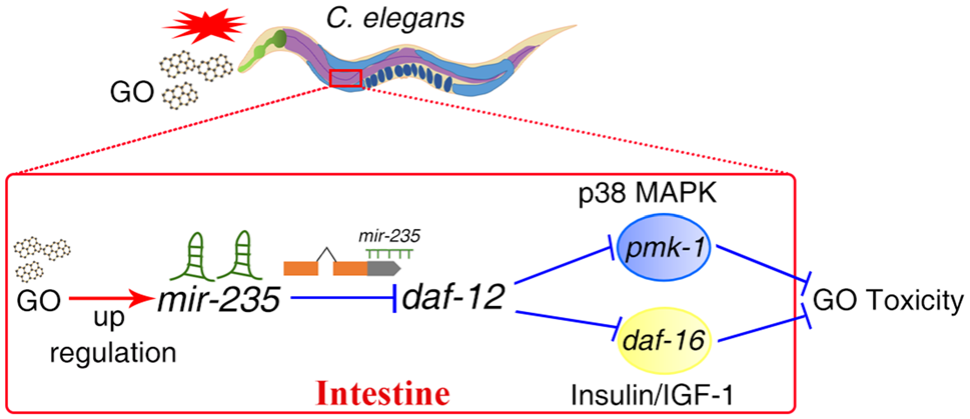
A diagram showing the intestinal *mir-235*-DAF-12-DAF-16 or *mir-235*-DAF-12-PMK-1 molecular signaling cascade involved in the control of GO toxicity in nematodes.

In conclusion, this study investigated that the potential molecular mechanisms of *mir-235* medicated the response to GO exposure in *C. elegans*. The intestinal specificity of *mir-235* in the regulation of GO toxicity was identified firstly. Importantly, we found that the *mir-235* affected the toxicity of GO by influencing the function of its target gene *daf-12* in the intestine. Furthermore, we demonstrated that DAF-16 in the insulin/IGF signaling pathway and PMK-1 in p38 MAPK signaling pathway acted the downstream of *daf-12* and play a key role in regulating GO toxicity in parallel. Therefore, our results indicate that *mir-235* mediates a protective mechanism against GO toxicity by suppressing the function of DAF-12-DAF-16 and DAF-12-PMK-1 signaling cascade in the intestine of nematodes (Fig. 9).

## Methods

### Reagents and preparation of GO suspensions

GO was prepared from a natural graphite powder according to the modified Hummer’s method^50^. First, graphite (2 g) and sodium nitrate (1 g) were added in a 250 mL flask. Next, the concentrated H_2_SO_4_ (50 mL) was added on ice. Then, KMnO_4_ (7 g) was added. After the temperature of the mixture reached 35 °C, 90 mL of deionized water was slowly dripped and stirred at 70 °C for 15 min to dilute the suspension. After treatment with a mixture of 7 mL of 30% H_2_O_2_ and 55 mL of deionized water, the suspension was filtered to obtain a yellow–brown filter cake. The filter cake was then washed three times with 3% HCl, and then dried at 40 °C for 24 h. Finally, GO would be obtained after the ultrasonication of as-made graphite oxide for 1 h.

GO was dispersed in K medium^51^ (50 mM NaCl, 30 mM KCl, 10 mM NaOAc, pH 6.0) to prepare the stock solution (1 mg/mL). The stock solution was sonicated for 30 min (40 kHz, 100 W) and diluted to the appropriate concentration with K medium before exposure. All the other chemicals were obtained from Sigma-Aldrich (St. Louis, MO, USA).

### Characterization of GO

GO was characterized by transmission electron microscopy (TEM, JEM-200CX, JEOL, Japan), atomic force microscopy (AFM, SPM-9600, Shimadzu, Japan), and Raman spectroscopy (Renishaw Invia Plus laser Raman spectrometer, Renishaw, UK) as previously described^40^. In addition, zeta potential was analyzed by the Nano Zetasizer using a dynamic light scattering technique (Nano ZS90, Malvern Instrument, Malvern, UK)^40^.

### Nematode strains and culture

The *C. elegans* strains of wild-type N2, NL2099[*rrf-3*(*pk1426*)], VP303[*rde-1*(*ne219*); *kbIs7*], MT17997[*mir-235*(*n4504*)], AA86[*daf-12*(*rh61rh411*)], JT204[*daf-12*(*sa204*)], CF1038[*daf-16*(*mu86*)], KU25[*pmk-1*(*km25*)] and the transgenic strain of TJ356 that carries a genome-integrated *daf-16::gfp* construct were obtained from Caenorhabditis Genetics Center(CGC). In addition, the transgenic strains *mir-235*(*n4504*)*Ex*(P*dpy-7-mir-235*), *mir-235*(*n4504*)*Ex*(P*unc-14-mir-235*), *mir-235*(*n4504*)*Ex*(P*ges-1-mir-235*),* Ex*(P*ges-1-mir-235*)* and Ex*(P*ges-1-daf-12-3′ UTR*) used in this study were constructed in the laboratory. Nematodes were maintained on nematode growth medium (NGM) plates seeded with *Escherichia coli* OP50 as food source at 20 °C^52^.

### GO exposure

Age synchronous populations of L1-larvae were obtained as previously described^53^. GO exposure was performed from L1-larvae for 96 h in 12-well sterile tissue culture plates in the presence of food at 20 °C^54^. The GO solutions were refreshed daily. The GO exposure concentration was 100 µg/L if not specially indicated.

### Locomotion behavior

Body bends and head thrashes were used as endpoints to evaluate the locomotion behavior^55,56^. After GO exposure, the nematodes were washed three times with M9 buffer, and were then transferred onto a freshly NGM plate without food to assay body bends or a freshly NGM plate without food but with 60 µL M9 buffer to assay head thrashes. A body bend is calculated as a change of posterior bulb direction, and a head thrash is defined as a change for bending direction at the mid body.

### Intestinal ROS production

The production of reactive oxygen species (ROS) was used to reflect the activation of oxidative stress and the functional state of the intestine^57^. To analyze ROS production, the examined nematodes were transferred to 12-well sterile culture plates with 1 mL M9 buffer containing 1 µM CM-H_2_DCFDA to pre-incubate for 3 h at 20 °C in the dark, and then mounted on 2% agar pads for examination with a laser scanning confocal microscope (Leica, TCS SP2, Bensheim, Germany) at a 488 nm excitation wavelength and a 510 nm emission filter. ROS production was semi-quantified by analyzing the fluorescent intensities, and expressed in relative fluorescent units (RFU).

### Bioinformatics analysis for candidate targeted gene prediction of *mir-235*

Bioinformatics software TargetScan version 6.2 (https://www.targetscan.org/worm_52/) was used to predict the possible targeted genes of *mir-235*. TargetScan is a tool for predicting miRNAs biological targets by finding conserved loci that match miRNA seed regions.

### Dissection of nematode intestines

To the extraction of *C. elegans* intestinal RNA, we dissected the intestine of nematodes as previously described^39^. Nematodes were picked into 20 µL M9 on a glass slide and carefully decapitated using a fine needle. Intestines were gently extracted and suspended in 50 µl of M9 in an Eppendorf tube. In total 250 intestines from each treatment were collected and processed for RNA extraction.

### Quantitative real-time polymerase chain reaction (qRT-PCR)

Total RNA was extracted using RNeasy Mini Kit (Qiagen). Approximately 6000 nematodes were used for each treatment. Total nematode RNA (~ 1 µg) was reverse-transcribed using cDNA Synthesis kit (Bio-Rad Laboratories). Quantitative reverse transcription polymerase chain reaction (RT-PCR) was performed at the optimal annealing temperature of 52 °C. The relative quantification of targeted genes in comparison to the reference *tba-1* gene encoding a tubulin protein was determined, and the final results were expressed as the relative expression ratio (between targeted gene and reference gene)^26^. The designed primers for targeted genes and reference *tba-1* gene are shown in Table S3.

### RNAi assay

RNAi assay was carried out by feeding nematodes with *E. coli* strain HT115 (DE3) expressing double-stranded RNA (dsRNA) homologous to the target gene^58^. *E. coli* HT115 (DE3) grown in LB broth containing ampicillin (100 μg/mL) was inoculated with ampicillin (100 μg/mL) and isopropyl β-d-thiogalactoside (IPTG, 5 mM) on NGM. L1 larvae nematodes were transferred to RNAi plates for 2 days at 20 °C until they developed into pregnancies. The pregnant adults were transferred to fresh RNAi- expressing bacterial lawns and allowed to lay eggs to obtain the second generation RNAi nematodes. Eggs were allowed to develop into young adults for subsequent assays. Primer information for RNAi is shown in Table S4.

### DNA constructs and germline transformation

To obtain vector carrying promoter sequence, promoter region for *ges-1* gene specially expressed in intestine, *unc-14* gene specially expressed in neurons, *dpy-7* gene specially expressed in hypodermis, was amplified by PCR from wild-type *C. elegans* genomic DNA. These promoter fragments were inserted into pPD95_77 vector. *mir-235* was amplified by PCR and inserted into corresponding entry vector carrying the *ges-1*, *unc-14* or *dpy-7* promoter sequence. Germline transformation was performed by co-injecting testing DNA (40 μg/mL) and marker DNA (P*dop-1::rfp*, 60 μg/mL) into the gonad of nematodes^59^. Primer information for vector construction is shown in Table S5.

### 3′-UTR reporters and microscopy

*daf-12* 3′ UTR (wt) was amplified by PCR from wild-type genomic DNA. *daf-12* 3′ UTR (mu) reporter was constructed by mutating the *mir-235* binding site in the *daf-12* 3′ UTR from GUGCAAU to GAACAAU. The 3′ UTR reporter containing a GFP vector driven by intestinal-specific *ges-1* promoter and GFP reporter under the control of the *daf-12* 3′ UTR (wild-type or *mir-235* binding site mutated) and P*ges-1*::mCherry *unc-54* 3′ UTR as a control were co-injected into the gonad of nematodes as described^59^. Primer information for vector construction is shown in Table S5. The fluorescence intensity of the first two pairs of intestinal cells was quantified by using Image J software.

### Distribution and translocation of GO

To detect the translocation and distribution of GO in nematodes, the fluorescent dye Rho B was loaded onto GO by incubating Rho B with an aqueous suspension of GO for 3 h as previous described^40–42^. Unbound Rho B was removed by dialysis against water over 72 h. After GO/Rho B exposure, nematodes were washed with M9 buffer for three times. The distribution of fluorescence in tissues of nematodes was observed under laser scanning confocal microscope. Triplicate independent experiments were performed, and thirty nematodes were examined for per treatment.

### Statistical analysis

Results were expressed as means ± standard deviation (SD) in this article. Figures were generated using GraphPad prism 7.00. All the data were analyzed using SPSS 12.0 software (SPSS Inc., Chicago, USA). Differences between groups were determined using analysis of variance (ANOVA). Probability levels between 0.05 and 0.01 were considered statistically significant.

## Supplementary information

Supplementary Information.
