# Supplementary material for: The C. elegans miR-235 regulates the toxicity of graphene oxide via targeting the nuclear hormone receptor DAF-12 in the intestine
Source: Sci Rep. 2020 Oct 9;10:16933. doi: 10.1038/s41598-020-73712-x (PMC7547681; doi:10.1038/s41598-020-73712-x)
Supplement: Supplementary file 1 — Supplementary Information. [file 41598_2020_73712_MOESM1_ESM.docx]

**The *C. elegans* miR-235 regulates the toxicity of graphene oxide via targeting the nuclear hormone receptor DAF-12 in the intestine**

Tiantian Guo^1^, Lu Cheng^1^, Huimin Zhao^1^, Yingying Liu^1^, Yunhan Yang^1^, Jie Liu^2^ and Qiuli Wu*^1^

1. Institute of Nephrology, Zhong Da Hospital, Medical School, Southeast University, Nanjing, China
2. Monash Biomedicine Discovery Institute and Department of Anatomy and Developmental Biology, Monash University, Melbourne, VIC 3800, Australia

Address correspondence to Qiuli Wu (qlwu@seu.edu.cn)

**Supporting Information:**


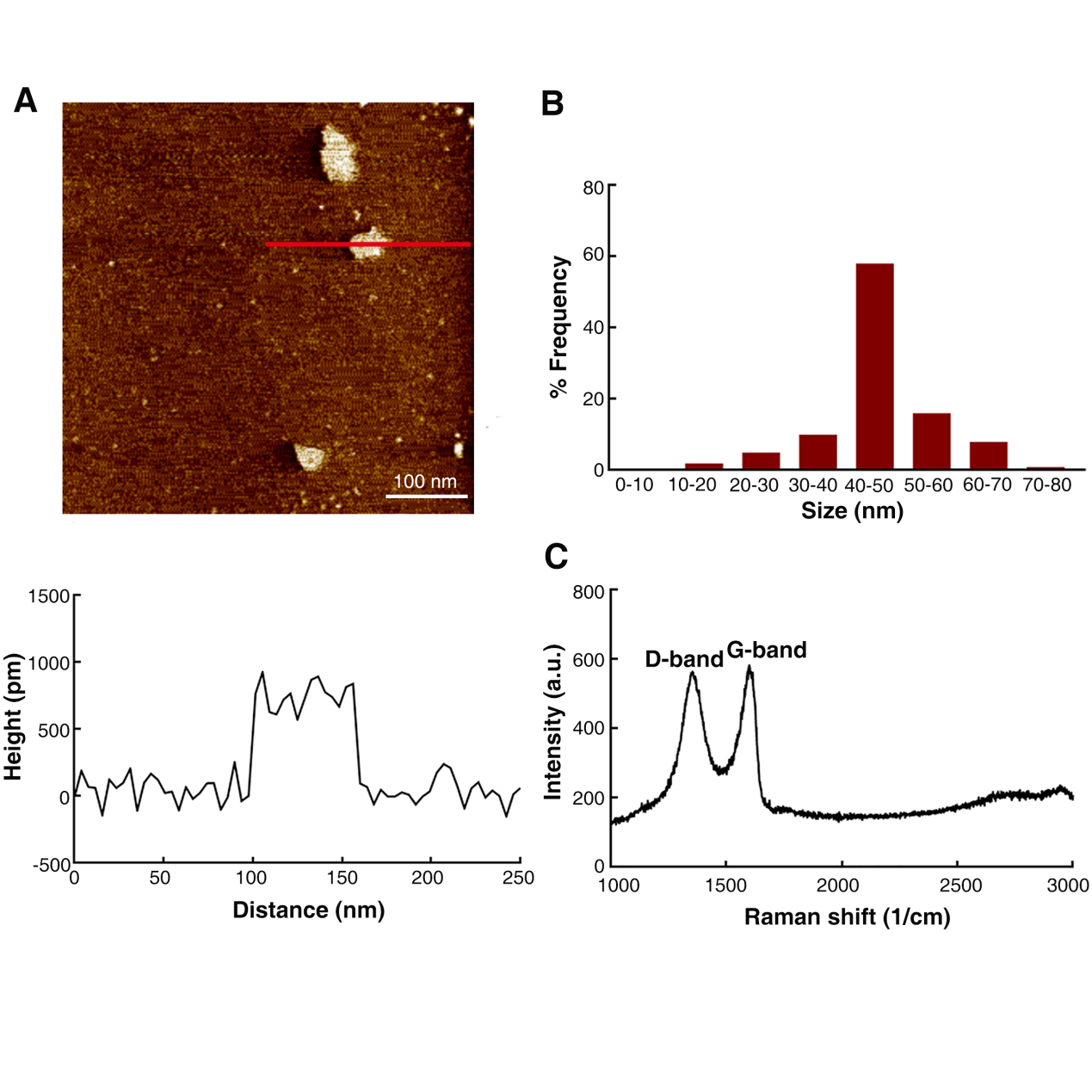


**Fig. S1.** Characterization of the GO. (A) AFM analysis of GO after sonification. (B) Size distribution of GO after sonification. (C) Raman spectrum of GO.

**
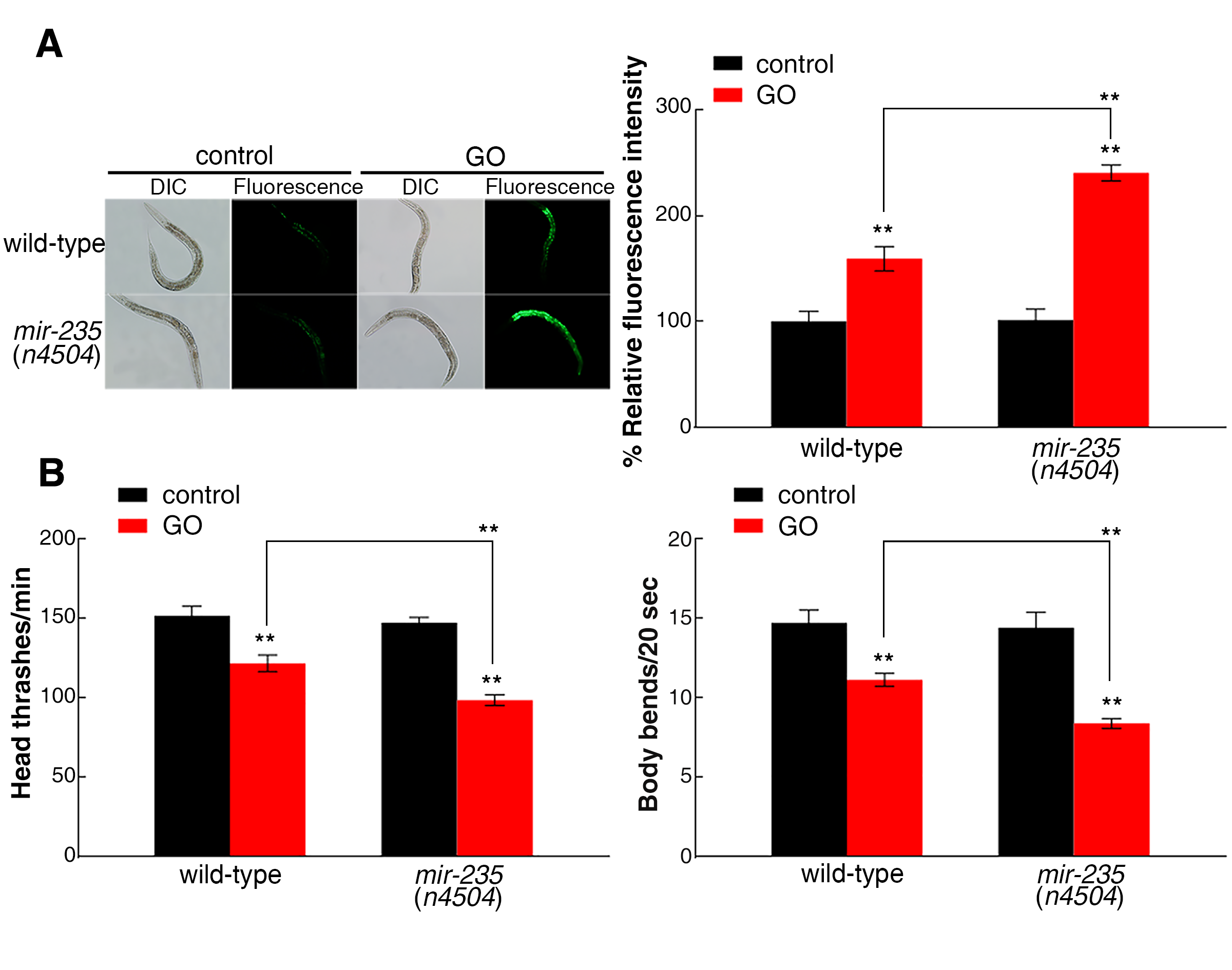
**

**Fig S2.** Effects of *mir-235* mutant nematodes on GO toxicity. (A) Effect of *mir-235* mutant nematodes on GO toxicity in inducing ROS production. (B) Effect of *mir-235* mutant nematodes in regulating GO toxicity in decreasing locomotion behavior. Bars represent means ± SD. ***P* < 0.01.


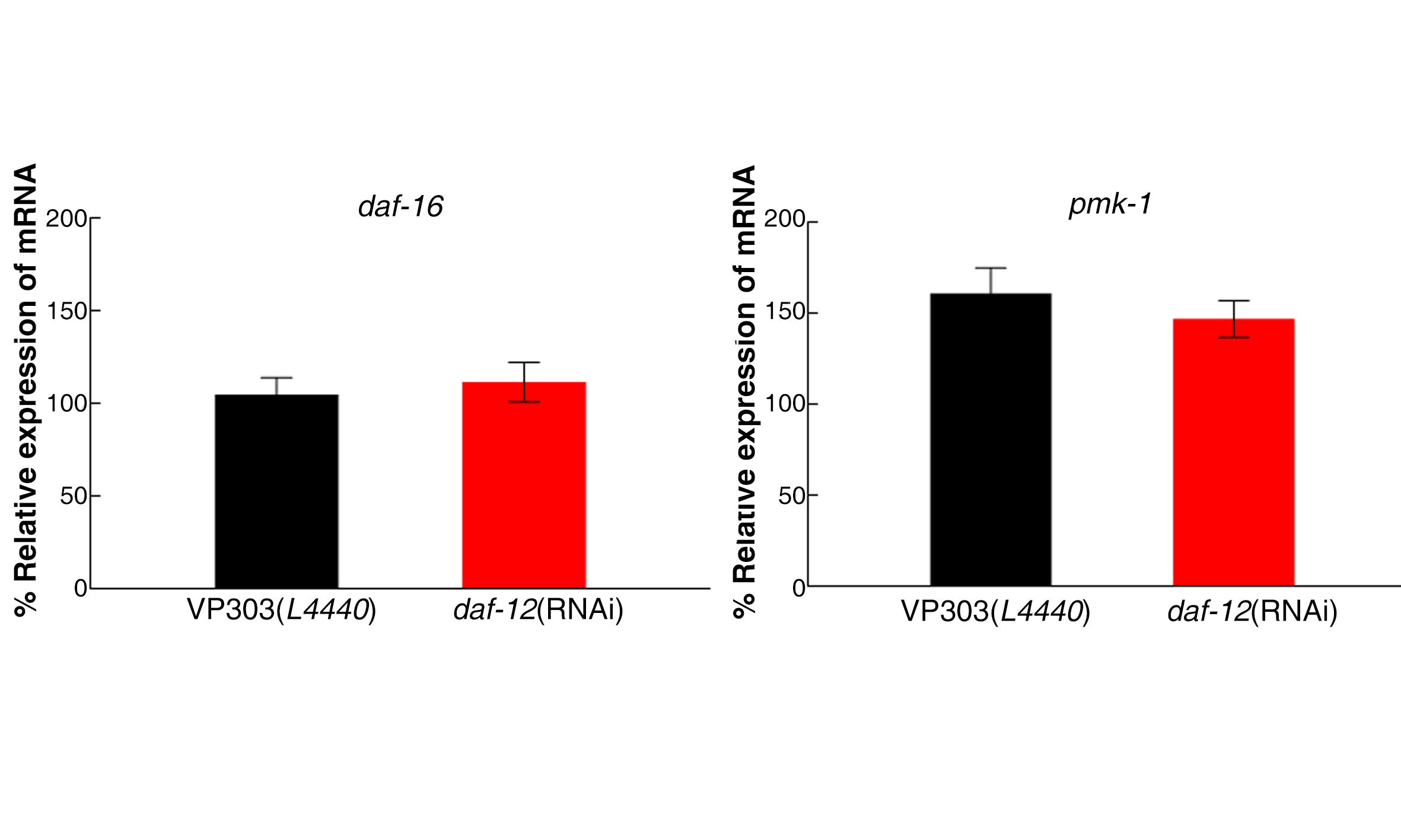


**Fig. S3.** Effects of *daf-16* and *pmk-1* expression without GO exposure in *daf-12* RNAi knockdown nematodes *via* qRT-PCR. Bars represent means ± SD.


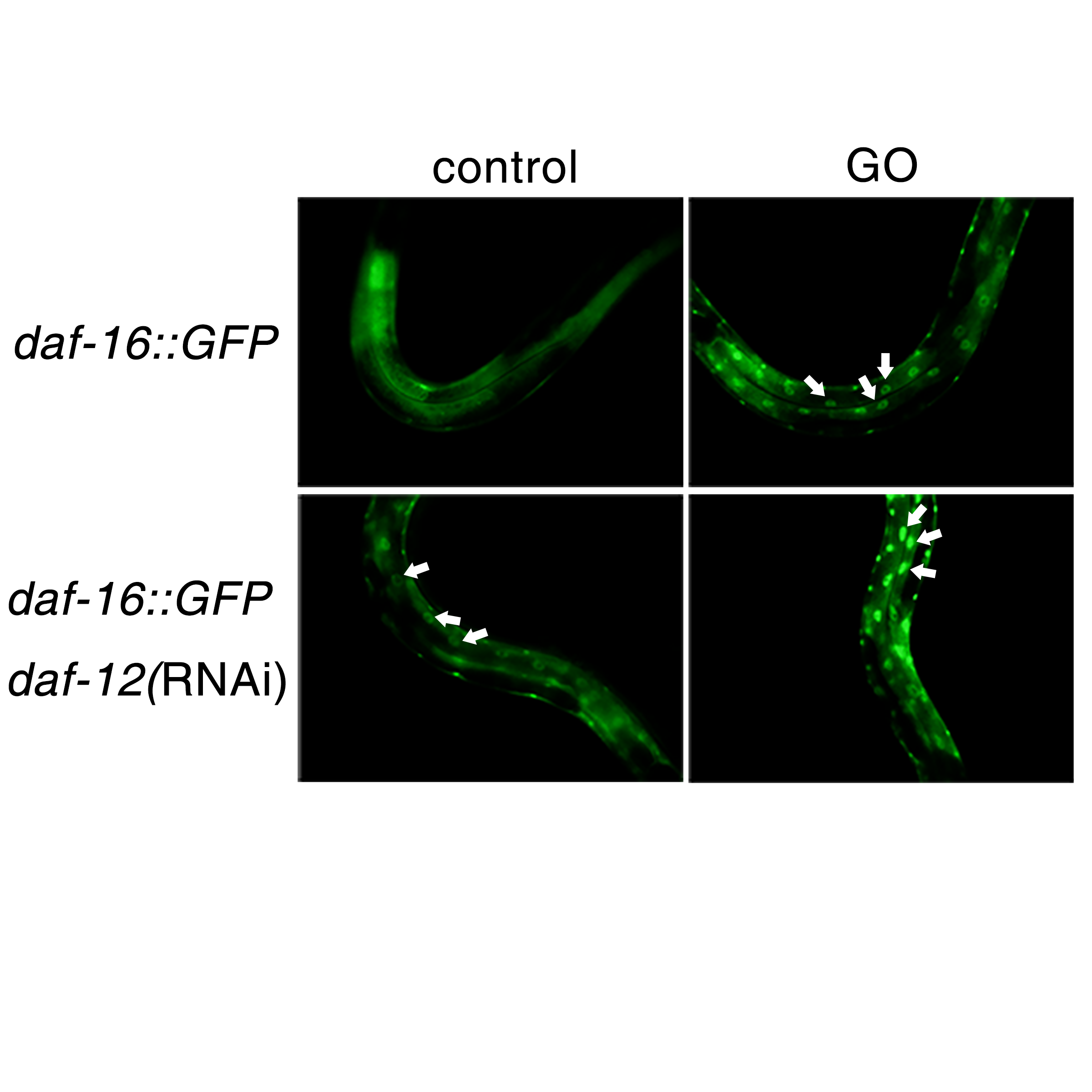


**Fig. S4.** Subcellular localization of DAF-16::GFP fusion protein in GO exposed *daf-12* RNAi knockdown nematodes. Bars represent means ± SD.


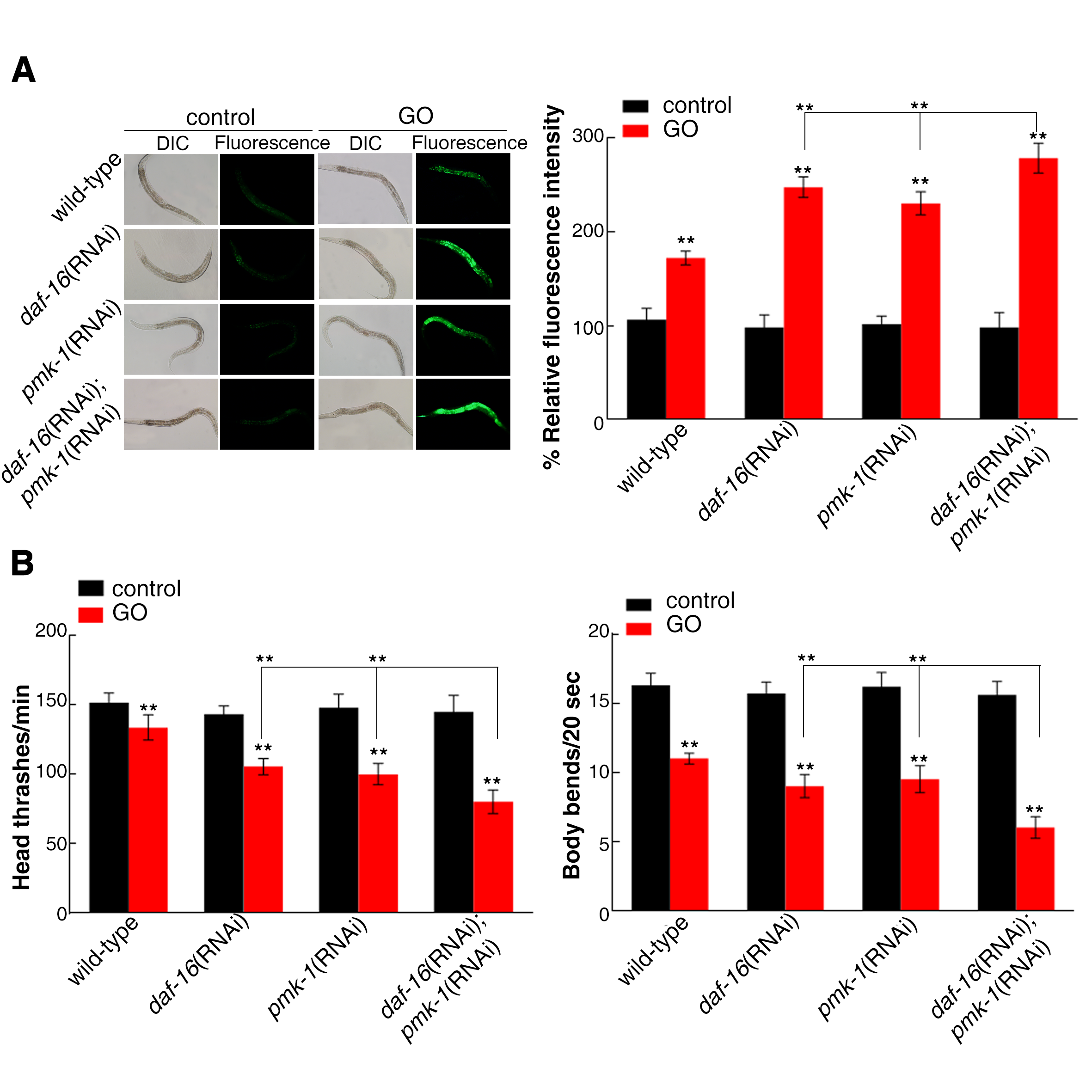


**Fig. S5.** Genetic interactions between *daf-16* and *pmk-1* in regulating GO toxicity. (A) Genetic interactions between *daf-16* and *pmk-1* in regulating GO toxicity in inducing ROS production. (B) Genetic interaction between *daf-16* and *pmk-1* in regulating GO toxicity in decreasing locomotion behavior in nematodes. Bars represent means ± SD. ***P* < 0.01.

**Table S1.** Candidate intestinal targeted genes of *mir-235*

| **Intestinal targeted genes** | | | | |
| --- | --- | --- | --- | --- |
| *abts-1* | *aex-3* | *alr-1* | *C06G8.1/swt-3* | *C34D4.4* |
| *C42C1.4* | *C52B9.4* | *cah-3* | *cwn-1* | *daf-12* |
| *din-1* | *eat-4* | *ets-4* | *F09F9.2* | *F27D9.2* |
| *F28C1.3* | *F40F8.1* | *F53F4.16* | *fkh-7* | *gpc-1* |
| *grl-7* | *ifc-2* | *jun-1* | *kin-18* | *mab-31* |
| *mca-3* | *mel-11* | *mls-2* | *myrf-1* | *nhr-25* |
| *nhr-71* | *nlp-9* | *nrfl-1* | *nstp-10* | *pdi-6* |
| *peb-1* | *plc-1* | *R09H10.5* | *rbc-1* | *sem-5* |
| *soap-1* | *stc-1* | *T14G11.1* | *T28D9.1* | *tag-97/svh-5* |
| *trxr-1* | *unc-11* | *unc-130* | *Y97E10C.1* | *zag-1* |

**Table S2.** The predicted binding site of *mir-235* to the 3' UTR of *daf-12* mRNA

|  | **predicted consequential pairing of target region (top) and miRNA (bottom)** | **seed match** | **conserved branch length** |
| --- | --- | --- | --- |
| Position 1308-1314 of *daf-12* 3’UTR  [*mir-235*](http://www.mirbase.org/cgi-bin/mirna_entry.pl?acc=cel-miR-235) |  | 7mer-m8 | 0.784 |

**Table S3.** Primer information for qRT-PCR of targeted genes of *mir-235*

| **Gene** | **Forward primer (5’-3’)** | **Reverse primer (5’-3’)** |
| --- | --- | --- |
| *rbc-1* | CACCATCATCCAAGGACATT | CTGTTGCATTTGGAATCGA |
| *ets-4* | CATGAACTCCCCTATTGACGCA | AGCAGGAGGGAAGACTCGATT |
| *C52B9.4* | ATGAAAACCGTAACCCTACT | TCTTTACTCTGTCTACGTGTAC |
| *grl-7* | CTGCTTCCTCAACCACTGCC | CATCAGAGTTGCAGAGTGGGT |
| *jun-1* | TCTATCGATGAAGATTCTGGCC | GGTTGTTGACGAAACGAAATTG |
| *mel-11* | CGGTAATTGATTCAAAACCCG | CGACATTGTTCATCTACTGCAA |
| *zag-1* | GCAAATTCAAGTGCCCGGAA | CCTGTTGCACGCATTTCTTCGA |
| *mca-3* | TGCACCGTTCTCATTCTCAT | GGCTCCGATGAATAGGCGAT |
| *F09F9.2* | TGCACCGTTCTCATTCTCAT | GGCTCCGATGAATAGGCGAT |
| *din-1* | TCGAGACACAACTCAATGTC | TTGATGAGTGGAGGTGGAAG |
| *peb-1* | AAGTTGAGCCACCATTCAGCCA | GGTGATCTTCCATTCCCATCCA |
| *F53F4.16* | GTATCACCTGGAACGGCC | ACGATTGCCGGATGTAG |
| *unc-130* | TCGAAAATCAATGTCTGGTC | GATTCATTGGATGTCCCAGC |
| *T14G11.1* | CACAATTTTTGGGAAGCCGT | TCAACGATCGGACAAACG |
| *C34D4.4* | CTTACACTACTATCAATGGACT | TCAGTGTTATTCCAACGG |
| *aex-3* | TGACAACACCCTCCGAACAC | GGCGAACGAGTGGATTGT |
| *nlp-9* | GGTCCATCTTCGCCACACCA | TGATCCAGCGAAGGCTCGT |
| *tag-97/svh-5* | AAGACACAACTCTTGATACTG | CGCATCTCTGATAAACTCC |
| *C06G8.1/swt-3* | TCGACAAATCTGGAAGAG | GAGATCCAACTCCATTTG |
| *T28D9.1* | GGTGAAAAGACAGAGGAGAC | CTCATCCTCAACCTTGCTT |
| *cah-3* | AAATGACCCCGGAGCTGCGA | GCTCCTCATTCGACAACGCCT |
| *alr-1* | TCAAAACAGACGAGCC | GCTACAACATCGGTGA |
| *abts-1* | GTAGAATCAGTTCCAAAGG | ATAACACCACAAAGTGCTC |
| *Y54E10A.16* | GATCGTGCAGAAAACGTCG | GATTCACATTCGGCACCTG |
| *trxr-1* | CGTGAGCGATACCCTCTCTA | CTTCCGGTAGTTTCCAACC |
| *unc-11* | TCCTTCCTCACCCGACTCGA | TGGGAAGGCACTTGGAGCT |
| *fkh-7* | CGTCCGCAGCAACCAACTCTA | TGAAAACGCAGCACCCTCA |
| *plc-1* | CAACATCACGTAGTGACTCA | GATTGTGGCATTCTCAGTG |
| *F28C1.3* | CTCCCCGATTCCACCAACA | GATGGTTGAGCACGAGCCA |
| *soap-1* | TGGATGAAACCCATAGTTT | AACTTGCGATCTTCACATT |
| *stc-1* | GGAAATAATCGTTTGGGAGGCC | TGTGGATGGAACATTCGTCAGC |
| *cwn-1* | GTGTTCGTTCGGGATC | GATTCGAGGTTTCTCAATA |
| *eat-4* | GCAAGAAGAAGGAAACGAA | CCAAGAAAGCAGATAAAGG |
| *ifc-2* | GAAGTGAATCCCAACCGACC | TGGGGGGTTTGGTCAGAA |
| *F27D9.2* | CTGGAACATAATGTAACGCA | CCATTTTCTTCATCTTGCACTA |
| *daf-12* | TGGTCATCCAATGCCAGC | GAGAGCCGATTGGATGCA |
| *R09H10.5* | ACGATTCAAAACTTTACTGC | TCTCTACAAAGTTCCATGTG |
| *tag-60* | AACAAAGGAACCCCAGATCAAG | GTGGATTCAATGGTGGAACATC |
| *C42C1.4* | CGACGTGCTACCGTAATCCGAC | AGGTGGAGGGTCAATGAGAGCT |
| *C50F4.14* | GATGCTCCGCTTTTCATCACTT | CCTTCCTGATCGACTCCTAACA |
| *kin-18* | GAACGTCGTCCACCATTGTTCA | GCTCCATTTCCTTCACTTCCTTC |
| *mls-2* | GGCATCTTCAGAGGATAGCACCAATG | GTCGGAAGTCCAAAGAATGGGAACAT |
| *nhr-71* | CAGTCCTGCCAAAGCAGTCCTA | TATCTTCCACCGGCAGTTCTCC |
| *F40F8.1* | TTGAACTACGTCCACTTATCAGCC | GGGAAGGTTTGTTGATTGTAGGTC |
| *sem-5* | CAACAAAGACGATCCAAACTGG | CGTTGGAGTTGCTCTTATTGCT |
| *gpc-1* | TTGCGAGAAGAACAAGACCAAC | GCTTTTCTTCTCTTGGAATGGA |
| *nhr-25* | GAGACCTGGATACTTAGTTGACCC | GGAAACAGTTGTCATTGGCATC |
| *pqn-47* | AGCTTCCGATACAATGCCACCA | CCGTTGAGGTTAGATGCCATTG |
| *tag-320* | GACAAAGAACTGCTCAGGCAAT | TCCTTCAAAGACCTCTGGAGCT |
| *Y97E10C.1* | TCTTCTTTGACATCTACCCTGGTC | ATTGGAAGCTTGATGGAAGAGC |
| *tba-1* | TCAACACTGCCATCGCCGCC | TCCAAGCGAGACCAGGCTTCAG |

**Table S4.** Primer information for RNAi of certain genes

| **Gene** | **Forward primer (5’-3’)** | **Reverse primer (5’-3’)** | **Restriction**  **enzyme site** |
| --- | --- | --- | --- |
| *daf-12* RNAi | AGATCTAGATCGCGTCAAG  GCTATC | ATACTGCAGCTCTTCGGCTTCACCA | XbaI/PstI |
| *daf-16* RNAi | AGACTGCAGGTACAGCAATTCCCAAATG | ACAGGTACCTGGATTTCGAAGAAGTGG | PstI/KpnI |
| *pmk-1* RNAi | CTGTCTAGAGTAACGGAGCCAATGTTTC | ATACCATGGGCGCCTAAATCCTCAAATCA | XbaI/NcoI |

**Table S5.** Primer information for vector construction

| **Gene** | **Forward primer (5’-3’)** | **Reverse primer (5’-3’)** | **Restriction enzyme site** |
| --- | --- | --- | --- |
| *Pdpy-7* | TACAAGCTTCTATGTGCAATGTCACGTGGA | CGCGGATCCCTGGAACAAAATGTAAGAATA | HindIII/BamHI |
| *Punc-14* | ACGAAGCTTTTCCCAACTGGCAATACT | TACTGCAGCCACAAAAGTTGAGAGCA | HindIII/PstI |
| *Pges-1* | ATATCTAGAAGCCACTCAGCCACTTCA | ATAGGATCCCATCTGAATTCAAAGATA | XbaI/BamHI |
| *Pdpy-7-mir-235* | ATACCCGGGTCCGAAGATATCAGGA | ATCGGTACCCGCCTTACTCTCAGAT | SmaI/KpnI |
| *Punc-14-mir-235* | ATACCCGGGTCCGAAGATATCAGGA | ATCGGTACCCGCCTTACTCTCAGAT | SmaI/KpnI |
| *Pges-1-mir-235* | ATAGGTACCGCTTCGCCTTACTCTCAG | ATGCCCGGGATCGAAACTCGTTTTCTT | SmaI/KpnI |
| *daf-12* 3’ UTR (wt) | ATACCCGGGACCTACTAGAAATCATCTACCAA | ATTGGTACCTGACAAGATACTGTTGGGGAGAA | SmaI/KpnI |
| *daf-12* 3’ UTR (mu) | ATACCCGGGACCTACTAGAAATCATCTACCAA | ATTGGTACCcattgTTctacacttttatttgt | SmaI/KpnI |
